# Loneliness, social support, and social networks: urban–rural variation and links to wellbeing in Scotland

**DOI:** 10.1007/s10389-024-02236-9

**Published:** 2024-03-25

**Authors:** Emily Long, Meigan Thomson, Jelena Milicev, Claire Goodfellow, Srebrenka Letina, Sara Bradley, Mark McCann

**Affiliations:** 1 MRC / CSO Social and Public Health Sciences Unit, University of Glasgow, Glasgow, G3 7HR UK; 2 Division of Rural Health & Well-Being, University of Highlands and Islands, Inverness, IV3 5SQ UK

**Keywords:** Urban, Rural, Social network, Wellbeing

## Abstract

**Aim:**

Social relationships are key public health priorities, with their relevance to wellbeing amplified in recent years. Relationships are embedded within the wider environment in which they occur; however, we lack understanding of whether, and how, places may affect social relationships. This study utilises an urban vs rural framework to examine variation in three specific aspects of relationships (loneliness, social support, and social networks), and their links to wellbeing.

**Subjects and method:**

Cross-sectional social network data, collected in Scotland in 2021 (*N* = 191), was used. Bivariate comparisons tested for differences in loneliness, social support, and social networks between urban and rural participants. Multivariable regression tested for associations between each construct and wellbeing, and interaction effects assessed differences in associations between the urban and rural locations.

**Results:**

Urban participants had higher levels of loneliness and poorer wellbeing, even though reported levels of social support didn’t differ. After adjusting for sociodemographic characteristics, loneliness, social support, and perceived emotional closeness of social networks were all associated with levels of wellbeing. There was no evidence that these associations differed between participants in urban and rural areas.

**Conclusion:**

Overall, findings highlight key place-based differences that inform the design of efforts to promote social connectivity and enhance wellbeing. Given that urban residents had lower wellbeing and higher levels of loneliness, coupled with evidence demonstrating the importance of close, supportive social relationships, intervention efforts that promote social connectivity in urban environments are particularly needed.

**Supplementary information:**

The online version contains supplementary material available at 10.1007/s10389-024-02236-9.

## Introduction

Social relationships are widely recognised as pivotal factors affecting health and wellbeing (Berkman et al. 2000; Latkin and Knowlton 2015; Montgomery et al. 2020). At the same time, concerns over loneliness and a lack of social connectivity have increased in recent years (Department for Digital, Culture, Media and Sport 2018; Victor et al. 2018), especially since Covid-19 and the related social restrictions (Groarke et al. 2020; Killgore et al. 2020).

People and places are intertwined, such that social relationships are likely to be impacted by the broader environment in which people live (Boessen et al. 2018; Small and Adler 2019; Walker and Hiller 2007). This is in line with the social ecological model, which posits that individuals are embedded within broader layers of the social environment, such as their social networks and communities, and that individual attributes interact with these wider systemic influences (Bronfenbrenner 2005; Sallis et al. 2008). Thus, in order to fully understand social relationships, and their associations with health, it is necessary to consider both person and place. To this end, place-based research into social connectivity and health is a key priority for public health policy (Department for Digital, Culture, Media and Sport 2018, 2022; Macdonald et al. 2021).

This study contributes to the literature by comparing three distinct aspects of social connectivity: loneliness, social support, and social networks, across two different types of places in Scotland: a large urban city, and a remote, rural area. Critically, the study links social connectivity (loneliness, social support, and social networks) to wellbeing, exploring differences across the urban–rural divide. In doing so, the study provides key insights for targeted policy and intervention efforts.

### Loneliness, social support, and social networks

Loneliness refers to the subjective perception of a discrepancy between desired and existing social relationships (Perlman and Peplau 1981), and is associated with myriad negative outcomes, including poorer physical health (Shankar et al. 2011; Valtorta et al. 2016), greater use of health care services (Valtorta et al. 2018), poorer mental health (Beutel et al. 2017; Wang et al. 2018), and even early mortality (Holt-Lunstad et al. 2015). Over 3 million adults in England report being lonely often or always (Department for Digital, Culture, Media and Sport 2021), with one in five adults living in Scotland reporting being lonely (Scottish Government 2020). As such, loneliness is increasingly recognised as a public health priority (World Health Organization 2021), with strategies to combat it enacted in the UK (Department for Digital, Culture, Media and Sport 2018), and Scotland specifically (Scottish Government 2018).

Social support is a broad concept encompassing support perceptions (perceived support) and receipt of supportive behaviours (received support), but only perceived support has been consistently strongly associated with health and wellbeing (Haber et al. 2007). Perceived social support, while related to loneliness, refers to people’s beliefs about how much support is available from their relationships, and the quality of this support (Dour et al. 2014; Hupcey 1998). Perceived social support is negatively associated with loneliness (Yildirim and Kocabiyik 2010), and positively with quality of life and psychological health (Wang et al. 2003), acting as a buffer against the negative effects of loneliness on mental health (Liu et al. 2016).^1^

At a broader level, the complex system of social relations in which people are embedded is conceptualized as a social network. The social network perspective focuses on the role of structural (e.g., how many people individuals interact with, whether their friends are friends with each other) and compositional characteristics of individual networks (attributes of network members such as age, gender, ethnicity, etc.). This knowledge is important, as there is evidence that network characteristics influence the availability and adequacy of social support, which, in turn, plays a role in a range of individual outcomes, including wellbeing (Borgatti et al. 2009; Scott and Carrington 2011).

### Rural–urban differences in social context

Framed by the social ecological model, urban and rural areas have distinct characteristics arising from their geographical, economic, and social differences, which can be associated with different patterns of settlement and may influence social relationships (Pateman 2011). The geographic isolation inherent in sparsely populated, scattered rural communities presents specific logistical challenges for delivering services and facilitating social connection. In particular, rural areas are more likely to have limited public transport (Henning-Smith et al. 2018) and poorer digital connectivity (Whitacre et al. 2017) than their urban counterparts, which are characterised by dense populations and greater proximity to services, facilities and opportunities for social interaction (Glaeser et al. 2001). Both rural and urban residents, however, experience social exclusion. While people from rural areas in South Yorkshire, England, reported lower levels of contact with friends, their urban counterparts experienced higher levels of threat and alienation in their local areas (Dahlberg and McKee 2018).

With marked differences in how supportive the environment could be for social relationships, there is the potential for urban–rural variability in loneliness, social support, and social networks. The majority of research investigating social relationships across urban and rural environments has focused on measures of social capital (a broad concept encompassing multiple features of relationships), generally finding higher levels of social capital in rural areas of the UK (Office for National Statistics 2016; Scottish Government 2020). Recent research has demonstrated higher levels of loneliness in more densely populated areas of the UK (Lai et al. 2021; Victor and Pikhartova 2020), further suggesting important place-based differences in the experiences of social relationships across urban and rural locations. Evidence for social support across urban and rural areas of the UK is lacking given the tendency for social support to be embedded within measures of social capital (Muller et al. 2021) precluding a separate examination of social support.

Research dating back several decades, predominantly conducted in the USA, has investigated social network characteristics of urban and rural residents, finding fewer family ties and more segmented networks in urban areas compared to rural locations (Fischer 1982; Hofferth and Iceland 1998; White and Guest 2003). More recent research, also based on US data (Cornwell and Behler 2015), demonstrated no differences in social network size or emotional closeness between urban and rural participants, but did find poorer neighbourhood quality to be associated with smaller, less close social networks for rural, but not urban, residents. However, extrapolating findings from US data to the UK context is problematic, given the vast differences in urban and rural typologies between the two countries (Pateman 2011). A clearer understanding of the extent to which key features of social networks, such as their structure (e.g., density) or composition (e.g., attributes of those in the network), differ between residents of urban or rural areas would inform intervention efforts that seek to leverage these relationships.

### Links with personal wellbeing

Personal wellbeing (PWB) is critical for public health due to its links to reduced use of health services (Keyes et al. 2010) and improved longevity (Diener et al. 2017; Diener and Chan 2011). Nevertheless, PWB is known to differ between urban and rural areas of the UK, with adults living in rural areas reporting greater wellbeing (Department for Environment, Food & Rural Affairs 2022; Hoogerbrugge and Burger 2021; Sørensen 2014). Studies that examined the relationship between social exclusion and PWB in England and Europe found that negative implications were stronger for urban residents (Dahlberg and McKee 2018; Spoor et al. 2014). This implies greater resilience of rural populations, potentially linked to the differences in loneliness, social network characteristics, and the support provided therein. To this end, evidence suggests that social relationships can be effectively targeted in efforts to improve PWB (Hunter et al. 2019; Umberson and Karas Montez 2010). Given that current public health initiatives encourage a social–ecological- and place-based approach to PWB (Public Health England 2021; Walker et al. 2021), and that loneliness and social connectivity are at the forefront of policy priorities (Department for Digital, Culture, Media and Sport 2018; Scottish Government 2018; van Woerden 2016; Victor et al. 2018), there is a need for better understanding of potential urban–rural differences in the links between social relationships and PWB.

As such, the current study explicitly examines urban–rural differences in associations between three dimensions of social connectivity (loneliness, social support, social networks) and PWB in order to provide specific, localized targets for interventions. Using data from two locations in Scotland, we first investigate whether there are differences in loneliness, social support, or characteristics of social networks between urban and rural residents (Research Question 1). We then assess the extent to which these aspects of social connectivity are associated with PWB, adjusted for demographics and urban/rural location (Research Question 2). Lastly, we test whether associations between loneliness, social support, or social networks and PWB differ between urban and rural participants (Research Question 3).

The study advances previous research by utilising UK-specific data to uncover how dimensions of social relationships vary across the urban–rural divide, including their associations with wellbeing. In doing so, the study provides new place-based insight into social connectedness in the UK, and identifies modifiable features of social relationships that could be leveraged in policy and intervention efforts to improve wellbeing.

## Materials and methods

### Participants and procedures

Data come from 191 adults (aged 16 and older) who took part in the Social Connections, Health, and Wellbeing in Scotland Study, conducted in 2021. The cross-sectional study aimed to depict the links between social connectedness and health in urban and rural locations in Scotland. The Scottish Government six-fold Urban–Rural Classification (Scottish Government 2022) was used to designate a ‘large urban area’ sample of participants and a ‘remote rural area’ sample. To maximise the difference in the features of the local environment, the ‘urban’ sample consisted of residents in Glasgow, which has a population greater than 125,000 residents (the highest category, 'Large Urban Areas', while the ‘rural’ sample consisted of participants who resided in communities of fewer than 3000 residents (the lowest category, 'Rural Areas' in the Scottish Highlands. For context, Glasgow is Scotland’s largest city (Population UK (2022), while the Highland Council area includes the most remote and sparsely populated parts of the UK, and has the lowest population density of the Scottish local authorities (i.e., regions; The Highland Council n.d.). Participants were recruited through postal mailers, randomised to the applicable postcodes corresponding to the sixfold classification and across the Scottish Index of Multiple Deprivation 2020 (Scottish Government n.d.). Participants were also recruited through stakeholder dissemination and social media. The survey was administered online between the months of April and July 2021. The study received ethical approval from the author-affiliated university and was performed in accordance with the ethical standards set out in the 1964 Declaration of Helsinki. All participants gave informed consent before taking part in the study.

### Measures

*Personal wellbeing (PWB)* corresponds to the Office for National Statistics recommended measure of personal wellbeing (Office for National Statistics 2018). As such, it was constructed as a composite measure based on three survey items: life satisfaction (‘Overall, how satisfied are you with your life nowadays’); happiness yesterday (‘Overall, how happy did you feel yesterday?’), and a sense that life is worthwhile (‘Overall, to what extent do you feel the things you do in your life are worthwhile?’). Each item was scored from 0 (not at all) to 4 (completely), and then summed to create a total score of wellbeing ranging from 0–12 (Cronbach’s α = 0.84), with higher scores indicating better wellbeing.

### Social network variables

Social network data was collected within the survey by asking participants to list the names and characteristics (e.g., gender) of individuals in their life. First, participants were asked to list up to 15 individuals whom they had spent time with or socialised with in the last month. Next, they were asked to list up to three additional people with whom they had not spoken in the last month, but still considered themselves connected to. The following social network variables were constructed from this data.

*Interaction network size* was calculated as the total number of individuals a participant indicated they had spent time with or socialized with in the last month, ranging from 0–15.

*Infrequent contact network size* was calculated as the total number of individuals a participant indicated they were connected to, but hadn’t spoken with in the last month. This variable ranged from 0–3.

*Total network size*. A measure of total network size was calculated by summing the number of individuals a participant named across both network questions, resulting in a variable ranging from 1–18.

*Closeness*. Participants were asked to indicate how close they felt to each person in their network ‘on a scale of 1 to 5, where 5 is very close and 1 is not very close.’ These scores were then averaged across each participant’s network, creating a closeness variable ranging from 1–5.

*Relationship diversity*. Participants were asked what their relationship was to each person in their network, with responses including spouse, partner/girlfriend/boyfriend, child, parent, other family, friend, colleague, neighbour, or other. A variable representing relationship diversity was then created using Shannon entropy (Shannon 1948), with higher scores representing greater diversity of relationship types in the network.

*Age similarity*. A variable representing the age similarity of participant’s social networks was created using the E-I index (Krackhardt and Stern 1988), which provides a relative measure of similarity between an individual and their social connections based on a specified attribute [e.g., age band (20–30 years, 31–40 years, etc.)]. The age similarity variable ranges from −1 to 1, with −1 indicating that all ties were the same age as the participant, and 1 indicating that no ties were the same age.

*Age diversity*. The diversity of ages within each participant’s network was calculated using Shannon entropy (Shannon 1948), with higher scores representing greater diversity of ages. While age similarity measures the similarity of a participant to their social connections, age diversity provides a measure of similarity within the network, but excluding the age of the participant.

*Proximity*. Participants were asked how physically close they lived to each person in their network, with responses ranging from 0 (live together) to 5 (they don’t live in the UK). These scores were averaged across each participants’ network to create a physical proximity measure ranging from 0–5.

*Density*. Participants were asked to indicate which of the individuals within their network knew each other. This data was used to measure density, representing the proportion of the possible connections in each participant’s social network that were actually present.

*Gender similarity*. A measure of gender similarity was created using the E-I index (Krackhardt and Stern 1988), resulting in a variable ranging from −1 to 1, with −1 indicating that all ties were the same gender as the participant, and 1 indicating that no ties were the same gender.

*Gender diversity*. The gender diversity within each participant’s network was calculated using Shannon entropy (Shannon 1948), with higher scores representing greater diversity of genders.

### Social support variables

*Social support*. A composite measure of social support was created from three items *(‘If I wanted company or to socialize, there are people I can count on’; ‘If I needed help, there are people who would be there for me’; ‘Is there anyone who you can really count on to listen to you when you need to talk?’*). The first two items were rated from 0 (definitely disagree) to 3 (definitely agree), while the last item was rated from 0–2 (‘No one’, ‘Yes, one person’, ‘Yes, more than one person’). The three items were summed (Cronbach’s α = 0.80) to create a scale from 0–8, with higher scores corresponding to more support.

*Frequency of socialising*. A 7-point scale of frequency of meeting up or talking with friends or family, ranging from never to at least once a day, was included, with higher scores reflecting more frequent socialising. This variable was included to provide a more objective measure of social interactions when assessing the subjective experience of perceived social support.

### Loneliness

*Loneliness* was assessed based on a single-item, direct measure of loneliness (‘How often do you feel lonely?’), to which participants responded from 1 (*never*) to 5 (*often/always*). The single-item, direct measure of loneliness is widely used in epidemiological studies of loneliness (Newmyer et al. 2021; Shiovitz-Ezra and Ayalon 2012). Higher scores indicate higher levels of loneliness.

### Demographic variables

The study controlled for demographic factors including *age* at time of survey completion, *location (‘*urban’ or ‘rural’), and *gender* (‘man’ or ‘woman’). The study also included binary indicators of whether a participant identified as *heterosexual,* was *British,* and whether they *live alone*. A measure of *perceived finances*, ranging from 0 (‘finding it very difficult’) to 4 (‘living comfortably’), a measure of *general health*, ranging from 0 (‘poor’) to 4 (‘excellent’), and a *broadband* connectivity variable, ranging from 0 (‘no access’) to 4 (‘very good access’) were also included.

### Data analysis

Three sets of analyses were conducted to answer our research questions. First, to detect differences in loneliness, social support, and social networks between participants in our urban and rural locations (Research Question 1), we conducted bivariate comparisons, using chi-squared tests for categorical variables and *t*-tests for continuous variables. Next, to assess how loneliness, social support, and social networks related to PWB (Research Question 2), we used a series of multiple linear regression models. A hierarchical approach was used, as this allowed for variables to be entered into the model based on each domain of interest (i.e., loneliness, social support, social networks). A full model, controlling for demographic variables and including relevant variables from each domain, was then tested. Lastly, to assess potential differences in predictors of PWB between the urban and rural locations (Research Question 3), interaction terms between location (i.e., urban/rural) and loneliness, social support, and social networks respectively were tested. Model assumptions for linear regression were tested, and no indications of multicollinearity [VIF  (variance inflation factor) < 0.10 and bivariate correlations < 0.80] were found. All analyses were conducted within R (R Core Team 2021). Complete case analysis was used to simplify modeling procedures.

## Results

Participants were 49 years old on average, and the sample was approximately 71% female. Participants from rural locations tended to be older, and were more likely to be female, heterosexual, and British. Participants in the rural location were also more likely to report poor broadband connectivity. See Table 1 for the full descriptive statistics of the sample.

**Table 1:** Sample characteristics by location

Parameter	Total sample	Urban	Rural	*P*-value
Sample size	191	81	110	
Age (mean, SD)	49.0 (15.9)	43.2 (16.0)	54.1 (14.1)	< 0.001
Gender (female)	70.7%	62.6%	78.0%	< 0.05
Heterosexual	84.3%	74.7%	93.0%	< 0.01
British ethnicity	90.6%	82.4%	98.0%	< 0.001
Live alone	30.4%	31.9%	29.0%	0.78
Employment status				0.06
Currently employed	62.3%	60.4%	64.0%	
Student	6.3%	12.1%	1.0%	
Retired	17.8%	14.3%	21.0%	
Perceived SES				0.64
Finding it very difficult	1.1%	2.2%	0.0%	
Finding it quite difficult	8.4%	8.8%	8.0%	
Just getting by	16.8%	17.6%	16.0%	
Doing alright	37.7%	36.3%	39.0%	
Living comfortably	35.6%	34.1%	37.0%	
Broadband				< 0.001
No access	2.6%	2.2%	3.0%	
Poor	9.4%	3.3%	15.0%	
Fair	20.4%	14.3%	26.0%	
Good	39.8%	40.7%	39.0%	
Very good	27.7%	39.6%	17.0%	
General health				0.46
Poor	6.3%	5.5%	< 1%	
Fair	16.2%	18.7%	14.0%	
Good	32.5%	26.4%	38.0%	
Very good	32.5%	35.2%	30.0%	
Excellent	12.6%	14.3%	11.0%	
Personal wellbeing (mean, SD)	7.52 (2.5)	6.96 2.4)	8.03 (2.4)	< 0.01

*p*-values are from two-tailed *t*-tests for continuous variables and chi-square tests for categorical variables

### Differences by location in loneliness, social support, and social networks

Results from the bivariate comparisons detecting differences in loneliness, social support, and social networks between the locations are displayed in Table 2. Rural participants reported lower levels of loneliness than their urban counterparts (*p* < 0.05), with rural participants reporting average loneliness scores of 1.60, compared to 2.05 for urban participants (range 0–4). No evidence of differences in social support or frequency of socialising was found.

**Table 2:** Loneliness, social support, and social networks by location

Parameter	Total sample	Urban	Rural	*P*-value
	Mean or %	Mean or %	Mean or %	
Loneliness	1.81	2.05	1.60	< 0.05
Social support	6.88	6.99	6.77	0.45
Meet up weekly	81.2%	79.1%	83.0%	0.62
Social networks				
Full network				
Total size	11.23	10.7	11.7	0.16
Interaction net size	9.43	9.13	9.70	0.38
Infrequent net size	1.80	1.55	2.03	< 0.05
Closeness	3.69	3.76	3.62	0.14
Diversity of ties	1.52	1.44	1.60	0.08
Gender EI	−0.21	−0.15	−0.26	0.11
Gender diversity	0.82	0.80	0.83	0.60
Age EI	0.23	0.13	0.33	< 0.01
Age diversity	1.58	1.42	1.72	< 0.001
Proximity	2.95	3.00	2.91	0.45
Density	0.43	0.41	0.45	0.26
Interaction network only				
Closeness	3.69	3.76	3.62	0.14
Diversity of ties	1.51	1.43	1.59	0.09
Gender diversity	0.82	0.81	0.83	0.68
Age diversity	1.57	1.41	1.71	< 0.001
Proximity	2.9	2.96	2.85	0.32
Density	0.46	0.41	0.50	< 0.05

*p*-values are from two-tailed *t*-tests for continuous variables and chi-square tests for categorical variables.

EI = equality index

Rural residents had a larger number of contacts in their peripheral network (2.03 in rural, 1.55 in urban, *p* < 0.05), and greater variation of ages within their social networks. Specifically, rural participants were less likely to be of a similar age to their social networks (*p* < 0.01), and more likely to have greater diversity of ages within their networks (*p* < 0.001). There were no differences across the remaining social network variables. A sensitivity test was conducted on the social network variables, in which we restricted the social networks to the interaction networks (i.e., the first 15 names), and re-ran each bivariate test. All parameters remained the same, with the exception of network density, which showed that participants from the rural locations had more densely connected networks (*p* < 0.05).

### Associations with PWB, and differences between urban and rural locations

Results from the regression analyses assessing loneliness, social support, and social network associations with PWB are displayed in Table 3. After adjusting for sociodemographic characteristics, urban residents had lower levels of PWB than rural residents (b = -−0.933, *p * < 0.01). Participants with better perceived finances had higher wellbeing (b = 0.928, *p* < 0.001), as did those with better physical health (b = 0.717, *p * < 0.001).

**Table 3:** Loneliness, social support, and social network associations with wellbeing

	Loneliness; coefficient (SE)	Social support; coefficient (SE)	Social networks; coefficient (SE)	Demographics; coefficient (SE)	Full model; coefficient (SE)
Loneliness	−1.158^***^ (0.131)				-0.505^***^ (0.144)
Support		0.696^***^ (0.104)			0.220^*^ (0.109)
Meet up		−0.055 (0.131)			0.020 (0.116)
Network size			0.207^***^ (0.058)		0.030 (0.035)
Closeness			0.687^*^ (0.287)		0.492^*^ (0.235)
Relationship diversity			0.700 (0.378)		0.096 (0.256)
Size of infrequent contact network			−0.206 (0.189)		
Age EI			0.410 (0.511)		
Age entropy			−0.473 (0.546)		
Proximity			0.137 (0.243)		
Density			1.049 (0.772)		
Gender EI			−0.271 (0.430)		
Gender entropy			−0.070 (0.721)		
Age category				0.025 (0.108)	−0.117 (0.104)
Location (Urban)				−1.161^***^ (0.323)	−0.933^**^ (0.304)
Gender (Male)				0.370 (0.331)	0.535 (0.323)
Heterosexual				−0.190 (0.449)	0.176 (0.433)
British				−0.765 (0.538)	
Live alone				−0.209 (0.313)	
Finances				0.928^***^ (0.173)	0.676^***^ (0.172)
General health				0.717^***^ (0.154)	0.426^**^ (0.153)
Digital connection				0.089 (0.147)	0.028 (0.142)
Constant	12.617^***^ (0.281)	6.181^***^ (0.684)	4.740^***^ (1.517)	7.837^***^ (1.071)	5.418^***^ (1.451)
*R*^2^	0.293	0.231	0.175	0.398	0.498
Adjusted *R*^2^	0.289	0.223	0.126	0.398	0.499

* *p* < 0.05; ** *p*  < 0.01; *** *p * < 0.001

EI = equality index

Loneliness was associated with poor PWB (b = -−0.505, *p* < 0.001), while higher social support (b = 0.220, *p* < 0.05) and higher emotional closeness of network ties were associated with better PWB (b = 0.492, *p * < 0.05). Though network size was linked with PWB in unadjusted models (b = 0.207, *p* < 0.001), this effect dissipated after fitting the full model.

Lastly, the interaction models showed no significant differences between participants in the urban and rural locations in the associations between loneliness (*p* = 0.60), social networks (*p * = 0.54), social support (*p* = 0.84), and PWB. See Supplementary Table 1 for the results from these models.

## Discussion

This study advances research by utilising UK-specific data to uncover how dimensions of social relationships vary across the urban–rural divide, including their associations with wellbeing. Our findings demonstrate differences in loneliness and aspects of social networks across urban and rural locations, but not in their associations with wellbeing. There was also strong evidence of poorer wellbeing among urban participants; and across all participants, those who reported greater loneliness demonstrated poorer wellbeing, while those with higher social support reported better wellbeing. Additionally, the emotional closeness of social network ties was related to wellbeing, but there was no evidence for the importance of other characteristics of social networks (e.g., size, density) for wellbeing.

While previous research has tended to focus on broad measures of social capital, finding higher levels of social capital among rural adults in the UK (Office for National Statistics 2016; Scottish Government 2020), we focused explicitly on three dimensions of social connectivity (i.e., loneliness, social support, social networks), each with important public health relevance. In line with the social capital literature and recent research on loneliness specifically (Lai et al. 2021; Victor and Pikhartova 2020), we found lower rates of loneliness among rural participants. Interestingly, levels of social support did not significantly differ between the urban and rural participants, suggesting that although urban participants experienced greater loneliness, this did not translate to reduced social support.

Moreover, several characteristics of social networks were found to differ across locations. Specifically, rural participants demonstrated greater variability of ages in their social networks, both in terms of their similarity in age to their social ties, as well as the age-similarity of their social ties with each other. This could be due to the smaller population of rural areas, limiting the social pool, and encouraging rural adults to seek social relationships with people in different life stages than themselves. Cross-generational familial relationships could also play a role due to potentially stronger links between long-established families in rural communities (Glass et al. 2020). Our findings align with research based on US data (Cornwell and Behler 2015), in that we did not find evidence of differences in social network size or emotional closeness with network members between participants from urban and rural areas. However, our study found differences in the size of participants’ infrequent contact networks, with rural participants indicating a larger number of people with whom they felt connected, but had not interacted with in the last month. Given that overall network size was not found to differ, this suggests that urban participants interacted with their social ties more frequently, whereas rural participants had a similar size of social circle, yet less frequent interactions with some social ties. Lastly, when considering only the interaction network (i.e., only social ties whom the participant had interacted with in the last month), rural participants were found to have more dense networks, in line with previous, older research on US samples (Fischer 1982). This finding is supported by literature asserting the close-knit nature of rural communities (Klärner and Knabe 2019).

Findings from the study demonstrate the relevance of all three relationship domains (loneliness, social support, and social networks) to PWB. Loneliness was significantly related to poorer wellbeing, in line with previous research (Goodfellow et al. 2022; Holt-Lunstad et al. 2015; Kearns et al. 2015; Tomaz et al. 2021), and suggesting that efforts to improve wellbeing in Scotland could benefit from targeting reductions in loneliness. In addition, social support was associated with increased PWB, whereas frequency of socialising with family or friends did not significantly relate to wellbeing. This suggests that time together does not confer the same wellbeing benefit as social support, indicating the key importance of relationship quality.

Mixed evidence was found for the role of social networks in wellbeing, with greater emotional closeness of social ties associated with higher PWB. Though network size was related to PWB in unadjusted models, this effect dissipated after controlling for loneliness, social support, and demographics. The remainder of the social network variables were non-significant, indicating that social network composition (e.g., age and gender diversity, relationship types), connectedness (i.e., density), and physical proximity to social ties were less important for wellbeing than emotional closeness of relationships.

Together, the findings highlight the relevance of social relationships to wellbeing, and offer malleable avenues for intervention. For example, intervention programs that focus on improving the exchange of support within relationships, or building trust and closeness, are more likely to experience increases in wellbeing than efforts that emphasize building more social connections or increasing the frequency of social contact. In addition, the findings highlight the importance of general physical health and finances to wellbeing, and these risk factors must be acknowledged in intervention efforts.

Lastly, our study found that wellbeing was lower amongst urban participants, in line with previous research from across the UK (Hoogerbrugge and Burger 2021). However, to the best of our knowledge, this is the first study to explicitly test whether associations between social relationships and wellbeing differed between urban and rural participants. To this end, we found no evidence of urban/rural variability in the effect of loneliness, social support, or social networks on PWB. The lack of significant interaction effects suggests that the role of place — in this case, residing in a rural area versus an urban city — does not significantly alter the relationship between loneliness, social support, or social networks and PWB. That is, regardless of whether a participant lived in a rural or urban area, loneliness remained a risk factor for poorer wellbeing, while a greater degree of social support and emotional closeness of network ties served as protective factors.

Several limitations of the study warrant mentioning. First, the study sample size is relatively small. Though participants were approximately evenly split between the two locations, and the sample size is large enough for simplistic models assessing cross-sectional associations, a larger sample size would allow for more complex modelling, and confidence in estimated coefficients. This is particularly true for our interaction effects, as the small sample size may have attenuated significance of effects. Secondly, the study is restricted to participants in Scotland, rather than the whole UK, limiting generalisability. Relatedly, we relied on binary indicators of ‘urban’ versus ‘rural’, precluding the assessment of differing effects across a continuum of rurality and urbanicity. As such, future research should seek to explore loneliness, social support, and social networks, including associations with wellbeing, across the whole of the UK, and using more finally grained measures of place (e.g., a spectrum of ‘rurality’). Also, the majority of participants were female, which could have affected estimates of gender homophily (i.e., gender equality index), for example. And finally, the data was collected during the timeframe of government restrictions regarding Covid-19 in the UK. Limitations on socialising in person may have impacted the findings. However, the two sample areas (i.e., urban, rural) were under similar restrictions during the timeframe of data collection, reducing the chance that Covid-related restrictions unequally impacted participants in the two areas.

Despite these limitations, the current study provides unique insight into place-based differences in three distinct aspects of social connectivity; loneliness, social support, and social networks. The study advances previous research by utilising UK-specific data to uncover how these vary across the urban–rural divide in Scotland, including their associations with wellbeing. The higher rates of loneliness and lower levels of wellbeing among urban participants, coupled with evidence demonstrating the importance of close, supportive relationships, suggest that intervention efforts that promote social connectivity in urban environments are particularly needed. Differences in social networks between urban and rural participants highlight varying social structure between areas, though we found no evidence that the links between loneliness, social support, and social networks and wellbeing differed across the urban/rural participants. As such, the study identifies modifiable features of social relationships that could be leveraged in policy and intervention efforts to improve wellbeing in both urban and rural environments.

## Supplementary information

Below is the link to the electronic supplementary material.

Supplementary file1 (DOCX 16 KB)

## Data Availability

Data will be made available through the UK Data Service ReShare, once the embargo period ends.

## Footnotes

1 From here on, this study uses the term *social support* to refer to *perceived social support*.

## Code availability

Code will be made publicly available through the Open Science Framework website https://osf.io.
