# Supplementary material for: Loneliness, social support, and social networks: urban–rural variation and links to wellbeing in Scotland
Source: J Public Health (Berl). 2024 Mar 25;33(12):2651–61. doi: 10.1007/s10389-024-02236-9 (PMC12630150; doi:10.1007/s10389-024-02236-9)
Supplement: Supplementary file 1 — Supplementary file1 (DOCX 16 KB) [file 10389_2024_2236_MOESM1_ESM.docx]

| **Appendix I. Results from interaction models** | | | |
| --- | --- | --- | --- |
|  | | | |
|  | Dependent variable: | | |
|  |  | | |
|  | Wellbeing | | |
|  | (Loneliness) | (Support) | (Social Networks) |
|  | *Coefficient, se* | *Coefficient, se* | *Coefficient, se* |
|  | | | |
| Location (urban) | -0.323 | -1.261 | 0.143 |
|  | (0.583) | (1.216) | (2.101) |
|  |  |  |  |
| Loneliness | -0.910^**^ |  |  |
|  | (0.406) |  |  |
|  |  |  |  |
| Location x Loneliness | -0.140 |  |  |
|  | (0.268) |  |  |
|  |  |  |  |
| Support |  | 0.610^**^ |  |
|  |  | (0.275) |  |
|  |  |  |  |
| Location x Support |  | 0.036 |  |
|  |  | (0.178) |  |
|  |  |  |  |
| Closeness |  |  | 1.154 |
|  |  |  | (0.864) |
|  |  |  |  |
| Location x Closeness |  |  | -0.342 |
|  |  |  | (0.560) |
|  |  |  |  |
| Constant | 13.031^***^ | 8.001^***^ | 7.937^**^ |
|  | (0.841) | (1.893) | (3.205) |
|  |  |  |  |
|  | | | |
| Observations | 191 | 187 | 187 |
| R^2^ | 0.307 | 0.273 | 0.073 |
| Adjusted R^2^ | 0.296 | 0.261 | 0.058 |
| Residual Std. Error | 2.072 (df = 187) | 2.128 (df = 183) | 2.406 (df = 183) |
| F Statistic | 27.666^***^ (df = 3; 187) | 22.922^***^ (df = 3; 183) | 4.818^***^ (df = 3; 183) |
|  | | | |
| Note: | ^*^p^**^p^***^p<0.01 | | |

Info for journal:

Title: Loneliness, social support, and social networks: Urban-rural variation and links to wellbeing in Scotland.

Journal: Journal of Public Health

Authors: Emily Long, Meigan Thomson, Jelena Milicev, Claire Goodfellow, Srebrenka Letina, Sara Bradley, & Mark McCann

Corresponding author: University of Glasgow, Emily.Long@glasgow.ac.uk
